# Positive End-Expiratory Pressure and Variable Ventilation in Lung-Healthy Rats under General Anesthesia

**DOI:** 10.1371/journal.pone.0110817

**Published:** 2014-11-10

**Authors:** Luciana M. Camilo, Mariana B. Ávila, Luis Felipe S. Cruz, Gabriel C. M. Ribeiro, Peter M. Spieth, Andreas A. Reske, Marcelo Amato, Antonio Giannella-Neto, Walter A. Zin, Alysson R. Carvalho

**Affiliations:** 1 Laboratory of Respiration Physiology, Carlos Chagas Filho Institute of Biophysics, Universidade Federal do Rio de Janeiro, Rio de Janeiro, Brazil; 2 Laboratory of Pulmonary Engineering, Biomedical Engineering Program, Alberto Luis Coimbra Institute of Post-Graduation and Research in Engineering, Universidade Federal do Rio de Janeiro, Rio de Janeiro, Brazil; 3 Pulmonary Engineering Group, Department of Anesthesiology and Intensive Care Medicine, Technische Universität Dresden, Germany; 4 Department of Anesthesiology and Intensive Care Medicine, University of Leipzig, Leipzig, Germany; 5 Cardio-Pulmonary Department, Pulmonary Division, Hospital das Clínicas, Universidade de São Paulo, São Paulo, Brazil; D'or Institute of Research and Education, Brazil

## Abstract

**Objectives:**

Variable ventilation (VV) seems to improve respiratory function in acute lung injury and may be combined with positive end-expiratory pressure (PEEP) in order to protect the lungs even in healthy subjects. We hypothesized that VV in combination with moderate levels of PEEP reduce the deterioration of pulmonary function related to general anesthesia. Hence, we aimed at evaluating the alveolar stability and lung protection of the combination of VV at different PEEP levels.

**Design:**

Randomized experimental study.

**Setting:**

Animal research facility.

**Subjects:**

Forty-nine male Wistar rats (200–270 g).

**Interventions:**

Animals were ventilated during 2 hours with protective low tidal volume (V_T_) in volume control ventilation (VCV) or VV and PEEP adjusted at the level of minimum respiratory system elastance (Ers), obtained during a decremental PEEP trial subsequent to a recruitment maneuver, and 2 cmH_2_O above or below of this level.

**Measurements and Main Results:**

Ers, gas exchange and hemodynamic variables were measured. Cytokines were determined in lung homogenate and plasma samples and left lung was used for histologic analysis and diffuse alveolar damage scoring. A progressive time-dependent increase in Ers was observed independent on ventilatory mode or PEEP level. Despite of that, the rate of increase of Ers and lung tissue IL-1 beta concentration were significantly lower in VV than in VCV at the level of the PEEP of minimum Ers. A significant increase in lung tissue cytokines (IL-6, IL-1 beta, CINC-1 and TNF-alpha) as well as a ventral to dorsal and cranial to caudal reduction in aeration was observed in all ventilated rats with no significant differences among groups.

**Conclusions:**

VV combined with PEEP adjusted at the level of the PEEP of minimal Ers seemed to better prevent anesthesia-induced atelectasis and might improve lung protection throughout general anesthesia.

## Introduction

Pulmonary atelectasis is a common complication during general anesthesia [1]. Anesthesia-induced atelectasis causes an increase in intrapulmonary shunt [2] and highly correlates with the progressive reduction in lung compliance and oxygenation impairment throughout surgery [1], [3]. Recruitment maneuvers have been used to open up atelectatic areas but their beneficial effects seemed to be just transitory and atelectasis rapidly recurs if no positive end-expiratory pressure (PEEP) is applied [4]–[8].

High levels of PEEP are often associated with decreased venous return [9], [10], increased pulmonary vascular resistance, decreased left ventricular compliance [11], [12], cardiac output and systemic oxygen delivery [13], [14], and still may result in lung overdistension with increased dead space ventilation and lung rupture [15]–[18]. In this line, the use of the so called open-lung PEEP [19] is still a challenge at the operating room routine [20], [21].

During controlled mechanical ventilation the breathing pattern is monotonous (tidal volume and respiratory rate are constant) and highly different from that observed in spontaneously breathing healthy subjects, which have an important intrinsic variability on tidal volume (V_T_) and respiratory rate (RR) [22]–[26]. Indeed, the addition of an extrinsic variability on ventilatory breathing pattern during mechanical ventilation, commonly referred to as variable ventilation (VV) [23], [26], appears to contribute to sustained improvement in gas exchange with reduced mechanical stress in several experimental models of acute lung injury [23], [27]–[29] and in lung-healthy patients during general anesthesia [30], [31]. Furthermore, the release of pro-inflammatory mediators (IL-6, IL-8, TNF-alpha) is significantly less in VV than in controlled ventilation [26], [29], [32].

Taking together, the combination of VV and PEEP may yield a better pulmonary function than protective low V_T_ strategies with comparable PEEP values [26]. We speculate that association VV and PEEP may result in increased alveolar stability, improved pulmonary function and attenuated inflammatory response in mechanically ventilated lung-healthy rats. Hence, we aimed at evaluating the physiological effects of the combination of VV and different levels of PEEP, adjusted after a recruitment maneuver and a decremental PEEP trial, on the improvement of alveolar stability and lung protection.

## Materials and Methods

Forty-nine male Wistar rats (200–270 g) were used in the present study. All animals received humane care in compliance with the “Principles of Laboratory Animal Care” formulated by the National Society for Medical Research and the “Guiding Principles in the Care and Use of Animals” approved by the Council of the American Physiological Society, USA. The experimental protocol was approved by the Ethics Committee on the Use of Animals, Health Sciences Centre, Federal University of Rio de Janeiro (approval number: IBCCF 103).

All animals were sedated (diazepam 5 mg, ip), anesthetized (ketamine, 60 mg/kg, ip), and intubated with a snugly fitting cannula (1.5 mm ID). Animals were placed in supine position on a surgical table and a catheter (18 G, Arrow International, USA) was inserted into the right carotid artery for continuous arterial pressure (AP) monitoring and blood sampling. At the end of surgical instrumentation, animals were paralyzed (pancuronium bromide, 0.3 mg/kg, iv) and mechanically ventilated (Inspira ASV, Harvard Apparatus, Holliston, MA, USA) using a volume controlled ventilation (VCV) mode, with a tidal volume (V_T_) of 6 mL/kg, respiratory rate (RR) of 90 breaths/min, inspiratory to expiratory time ratio (I:E) of 1∶2, positive end-expiratory pressure (PEEP) of 0 cmH_2_O and fraction of inspired oxygen of 0.5 (Baseline).

### Ventilation Protocol

After 5 minutes of stabilization period under baseline settings, lungs were recruited by increasing PEEP from 0 to 3, 6, 7 and 8 cmH_2_O, 30 seconds per step. Just afterwards, PEEP was progressively reduced from 8 to 0 cmH_2_O, in steps of 1 cmH_2_O for 30 s per step and the PEEP of minimum respiratory system elastance (PEEP_minErs_) was determined.

To investigate the physiological effects of each ventilation strategy (VCV or VV) combined with 3 different levels of PEEP, ventilation lasted 2 hours with the same baseline settings but with: 1) VCV or VV with mean V_T_ of 6 mL/kg and PEEP adjusted at the PEEP_minErs_ (VCV_E or VV_E, respectively n = 7 per group); 2) VCV or VV with PEEP adjusted at the PEEP_minErs_ plus 2 cmH_2_O (VCV_E+2 or VV_E+2, respectively n = 7 per group); and 3) VCV or VV with PEEP adjusted at the PEEP_minErs_ minus 2 cmH_2_O (VCV_E-2 or VV_E-2, respectively n = 7 per group).

**Figure 1:**
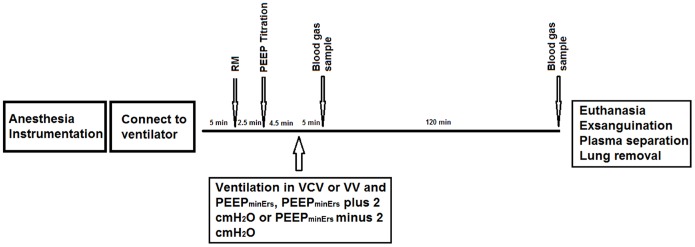
Time course of respiratory system elastance and slope. **A.** Respiratory system elastance (Ers) throughout ventilation protocols. Red points are minute-averaged values of Ers and its fitted line (blue line). **B.** Slope of Ers calculated by angular coefficient of the groups: VCV_E (volume-controlled mode and PEEP_minErs_; n = 6), VCV_E+2 (volume-controlled mode and PEEP_minErs_ plus 2 cmH_2_O; n = 6), VCV_E-2 (volume-controlled mode and PEEP_minErs_ minus 2 cmH_2_O; n = 6), VV_E (variable ventilation and PEEP_minErs_; n = 6), VV_E+2 (variable ventilation and PEEP_minErs_ plus 2 cmH_2_O; n = 5), VV_E-2 (variable ventilation and PEEP_minErs_ minus 2 cmH_2_O; n = 6). Effect of ventilator mode, PEEP and interaction were tested with two-way ANOVA followed by Tukey post-hoc. Time effect was tested by generalized linear model. Statistical significance was accepted at p<0.05. *p<0.05.

Fig. 1 illustrates the time course of the experimental protocol. Seven animals were surgically instrumented and immediately sacrificed (Fig. 1) to be used as controls for protein assays and histology.

### Variable Ventilation Design

VV mode was designed to provide different range of V_T_ adjusted to achieve a desired target variability level. The widths of the V_T_ distributions were set by assigning a random sequence of V_T_ values taken from a Gaussian probability distribution falling between ±10% of the mean V_T_ (6 mL/kg). A sequence of 5400 values of V_T_ was applied during the first hour and repeated in the second hour of the protocol. Thus, the measured mean and ± one SD was 5.9±0.8 mL/kg. RR was kept constant throughout the protocol as previously proposed [26].

### Data Acquisition and Processing

Airway pressure (Paw) and flow (F) were continuously recorded using a heated-controlled pneumotachograph (Hans Rudolph model 8430B, Shawnee, KS, USA) connected between the endotracheal tube (ETT) and a Y-piece of the ventilatory circuit. The pneumotachograph was connected to a differential pressure transducer for Paw (105124-9, SCIREQ, Montreal, QC, Canada) and flow (105159-6, SCIREQ, Montreal, QC, Canada) measurements. Arterial blood pressure as well as the electrocardiogram was continuously acquired with a Multi-parameter physiologic monitor (LifeWindow LW6000 Digicare Biomedical Technology, Boynton Beach, FL, USA). Paw and flow signals were low-pass filtered at 30 Hz and all signals were then digitized at 1000 Hz using a 16-bit AD converter (NI-6009, National Instruments, Austin, TX, USA) and recorded with a built-purpose routine (Data Acquisition System, DAS) written in LabVIEW (National Instruments, Austin, TX, USA). Volume was calculated by numerical integration of flow. All transducers were calibrated before the experiments.

During the entire protocol, respiratory system elastance (Ers) was estimated in real-time by means of the recursive least square method (5 s forgetting time constant) considering the linear single-compartment model (Equation 1) in a subroutine of the DAS software.



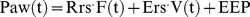
(1)Where Rrs and Ers represent the resistance and elastance of the respiratory system, respectively, and EEP is the total end-expiratory pressure at null volume (V) and flow (F).

After the experiments, all mechanical parameters were also calculated offline on a breath-by-breath basis for the whole 120 minutes of ventilation period. For such purpose the least square method was applied to Equation 1 and a routine (Mecanica) written in MATLAB (The Mathworks Inc., Natick, MA, USA) was employed. The mean Ers was calculated for every 1-minute period, and the temporal dynamics of Ers was assessed by the slope (a) of a linear function described by Ers (t) = a.t+b; where t is time in minutes and b is the Ers at the beginning of ventilation protocols.

Mean arterial pressure (MAP) was also calculated offline as the mean value of arterial pressure beat-by-beat throughout the experimental protocols.

Arterial blood samples were anaerobically collected and immediately processed to measured PaO_2_, PaCO_2_ and pH (i-STAT, Abbott Laboratories, Abbott Park, IL, USA), at 5 (M5) and 120 minutes (M120) of the ventilation protocols.

### Digital histology image acquire and processing

At the end of the experimental protocol, mechanical ventilation was returned to baseline settings. After heparinization the animals were euthanized by sectioning the abdominal vena cava. The trachea was clamped at end-expiration and the lungs were extracted *en bloc*, the right and left lungs were isolated and fixed in formaldehyde 10% in Millonig’s phosphate buffer (100 ml HCHO, 900 ml H2O, 18.6 g NaH2PO4, and 4.2 NaOH) and subsequently embedded in paraffin. 4-µm thick transversal slices were cut from the apex to the base of the left lung and stained with hematoxylin-eosin.

**Figure 2:**
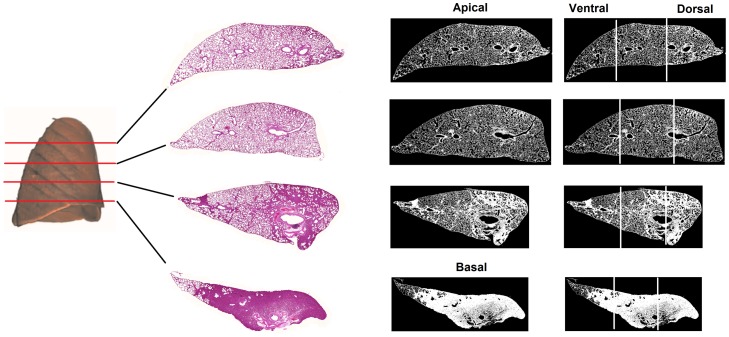
Air fraction obtained by histology images. Air fraction analysis of apex and base (A) and ventral and dorsal lung areas (B) in the groups: VCV_E (volume-controlled mode and PEEP_minErs_; n = 6), VCV_E+2 (volume-controlled mode and PEEP_minErs_ plus 2 cmH_2_O; n = 6), VCV_E-2 (volume-controlled mode and PEEP_minErs_ minus 2 cmH_2_O; n = 6), VV_E (variable ventilation and PEEP_minErs_; n = 6), VV_E+2 (variable ventilation and PEEP_minErs_ plus 2 cmH_2_O; n = 5), VV_E-2 (variable ventilation and PEEP_minErs_ minus 2 cmH_2_O; n = 6). Ventilatory mode and PEEP effects were tested with two-way ANOVA followed by Tukey post hoc. t test was used to detect differences between ventilated and non-ventilated groups and then correction by comparisons ensued. Statistical significance was accepted at p<0.05. *p<0.05.

The histologic lung slices were then digitized (3DHistech Panoramic Scan, Budapest, Hungary) and images were processed using a routine written in MATLAB (MathWorks, Inc., Natick, MA, USA) in order to quantify the fraction of air in each specimen. The specimen images were converted to binary images, the total specimen area was computed and the relative proportion between the amount of parenchyma, edema or infiltration (presented in white color) and air (presented in black) were determined in each specimen (Fig. 2).

Diffuse alveolar damage (DAD) was evaluated using an histo-pathological scoring system by an expert blinded to the [33]. Briefly, five typical features of DAD were evaluated, according to their extent and severity. Single features, namely alveolar edema, interstitial edema, inflammatory infiltration, hemorrhage and micro-atelectasis were scored ranging from 0–9 and the cumulative DAD score, ranging from 0–45, was calculated by summarizing the single score features.

### Protein assays

The right lung homogenates and plasma samples collected at the end of ventilation protocols were used for the assessment of pro-inflammatory (TNF-α, IL-1β, IL-6, CINC-1) mediators with the ELISA technique, with high sensitivity kits (R&D Systems Inc., Minneapolis, MN, USA) according to the manufacturer’s instructions.

### Statistical analysis

Statistical analysis was performed with SigmaPlot 11 (Systat, Chicago, IL, USA). The normality of the data (Kolmogorov-Smirnov test with Lilliefors correction) and the homogeneity of variances (Levene median test) were tested. Then, Two-way ANOVA (mode and PEEP and their interactions as independent factors) was used. For *post-hoc* analyses, we used Tukey pairwise comparison. To compare non-ventilated and ventilated groups, the t-test was performed followed by the Bonferroni multiple comparison correction. A general linear model for repeated measures was used to evaluate the influence of time on deterioration of Ers throughout each ventilation protocol. All tests considered a critical *p<*0.05.

## Results

Ventilatory and hemodynamic data are shown in Table 1. Tidal volume (V_T_), respiratory rate (RR), mean arterial pressure (MAP) and heart rate (HR) did not differ among groups. PEEP_minErs_, determined during the decremental PEEP trial, did not differ among groups, ranging between 3 to 5 cmH_2_O. The coefficient of variation of V_T_ was significantly higher, as expected, in VV groups compared to VCV (8.5±0.5% *vs* 1.6±0.6%, respectively; p<0.001). Mean airway pressure (Pmean), peak airway pressure (Ppeak) and positive end-expiratory pressure (PEEP) were significantly different among PEEP settings (p<0.0001).

**Table 1:** Ventilatory and hemodynamic variables.

Group	VCV_E-2	VV_E-2	VCV_E	VV_E	VCV_E+2	VV_E+2	Mode	PEEP	Interaction
**Ppeak (cmH_2_O)**	11±1.6	12±1.3	14±0.5	13±1.3	15±0.8	14±1.3	ns	**	ns
**Pmean (cmH_2_O)**	5±0.8	5±0.7	6±0.6	6±0.7	9±0.6	8±0.7	ns	**	ns
**PEEP (cmH_2_O)**	[1]–[2]	[1]–[2]	[3]–[4]	[3]–[4]	[5]–[7]	[5]–[6]	ns	**	ns
**Mean V_T_ (mL/kg)**	5.8±0.2	6.1±0.7	5.5±0.3	5.7±0.2	5.6±0.4	6.0±1.1	ns	ns	ns
**RR (bpm)**	84±0.6	84±0.6	84±1.4	84±0.2	84±0.4	84±0.2	ns	ns	ns
**CVV_T_ (%)**	1±0.2	8±1.0	2±0.5	8±0.4	1±0.3	10±3.3	**	ns	ns
**MAP (mmHg)**	110±15	120±21	101±28	97±23	118±24	115±19	ns	ns	ns
**HR (bpm)**	410±65	389±38	383±68	406±32	402±70	393±57	ns	ns	ns

Values are mean ± SD from initial 5 minutes of ventilation. PEEP values are given as range (minimum and maximum). Definition of abbreviations: VCV_E = volume-controlled mode and PEEP_minErs_; VCV_E+2 = volume-controlled mode and PEEP_minErs_ plus 2 cmH_2_O; VCV_E-2 = volume-controlled mode and PEEP_minErs_ minus 2 cmH_2_O; VV_E = variable ventilation and PEEP_minErs_; VV_E+2 = variable ventilation and PEEP_minErs_ plus 2 cmH_2_O; VV_E-2 = variable ventilation and PEEP_minErs_ minus 2 cmH_2_O; BW = body weight; Ppeak = peak airway pressure; Pmean = mean airway pressure; PEEP = positive end-expiratory pressure; V_T_ = tidal volume; RR = respiratory rate; CVVT = tidal volume coefficient of variation; MAP = mean arterial pressure; HR = heart rate. Differences among groups were tested with two-way ANOVA. Statistical significance was accepted at p<0.05. **p<0.0001, ns: non-significant.

**Figure 3:**
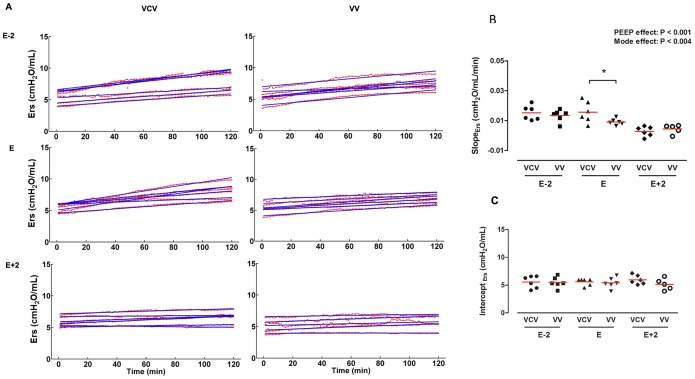
Lung cytokines. Lung tissue concentration of TNF-α (A), IL-6 (B), CINC-1(C) and IL-1β(D) in the groups: VCV_E (volume-controlled mode and PEEP_minErs_; n = 6), VCV_E+2 (volume-controlled mode and PEEP_minErs_ plus 2 cmH_2_O; n = 6), VCV_E-2 (volume-controlled mode and PEEP_minErs_ minus 2 cmH_2_O; n = 6), VV_E (variable ventilation and PEEP_minErs_; n = 6), VV_E+2 (variable ventilation and PEEP_minErs_ plus 2 cmH_2_O; n = 5), VV_E-2 (variable ventilation and PEEP_minErs_ minus 2 cmH_2_O; n = 6). Red lines represent mean values. Ventilatory mode and PEEP effects were tested with two-way ANOVA followed by Tukey post hoc. T-test was used to detect differences between ventilated and non-ventilated groups and then correction by multiple comparison. Statistical significance was accepted at p<0.05. *p<0.05.

The dynamics of Ers in each animal at each ventilation protocol is represented in Figure 3A. In all groups, Ers depended on time (p<0.0001) and always increased throughout ventilation period. The rate of change of Ers(t) (slope) clearly decreased with PEEP both in VCV or VV (p<0.001). However, Ers(t) slope in the VV mode and PEEP_minErs_ was significantly lower than in VCV (0.016±0.007 vs 0.009±0.002 cmH_2_O/L/min, respectively; p<0.001), thus suggesting better Ers stability (Fig. 3B).

**Figure 4:**
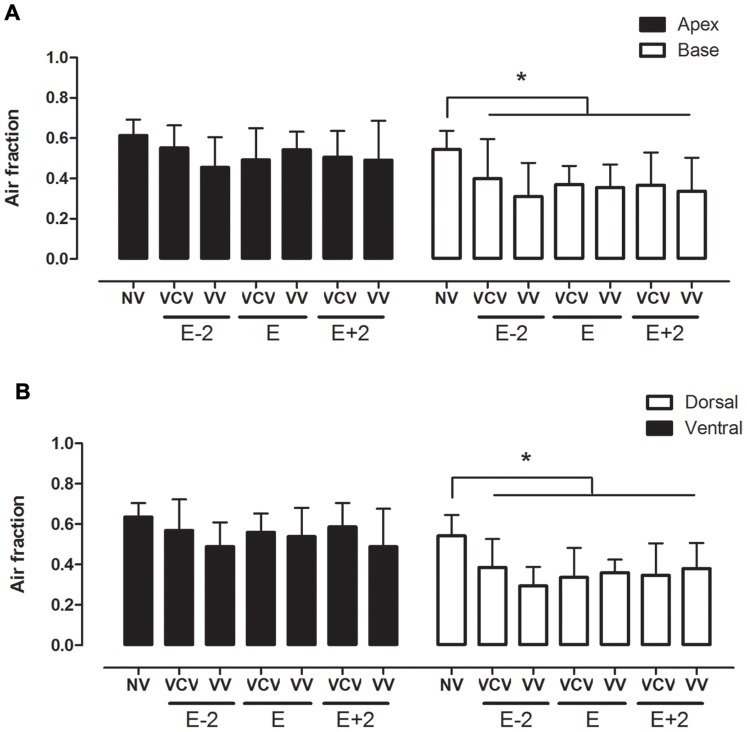
Plasmatic cytokines. Plasmatic concentration of TNF-α (A), IL-6 (B), CINC-1(C) and IL-1β (D) ) in the groups: VCV_E (volume-controlled mode and PEEP_minErs_; n = 6), VCV_E+2 (volume-controlled mode and PEEP_minErs_ plus 2 cmH_2_O; n = 6), VCV_E-2 (volume-controlled mode and PEEP_minErs_ minus 2 cmH_2_O; n = 6), VV_E (variable ventilation and PEEP_minErs_; n = 6), VV_E+2 (variable ventilation and PEEP_minErs_ plus 2 cmH_2_O; n = 5), VV_E-2 (variable ventilation and PEEP_minErs_ minus 2 cmH_2_O; n = 6). Red lines represent mean values. Ventilatory mode and PEEP effects were tested with two-way ANOVA followed by Tukey post hoc. T-test was used to detect differences between ventilated and non-ventilated groups and then correction by multiple comparison. Statistical significance was accepted at p<0.05. *p<0.05.

**Figure 5:**
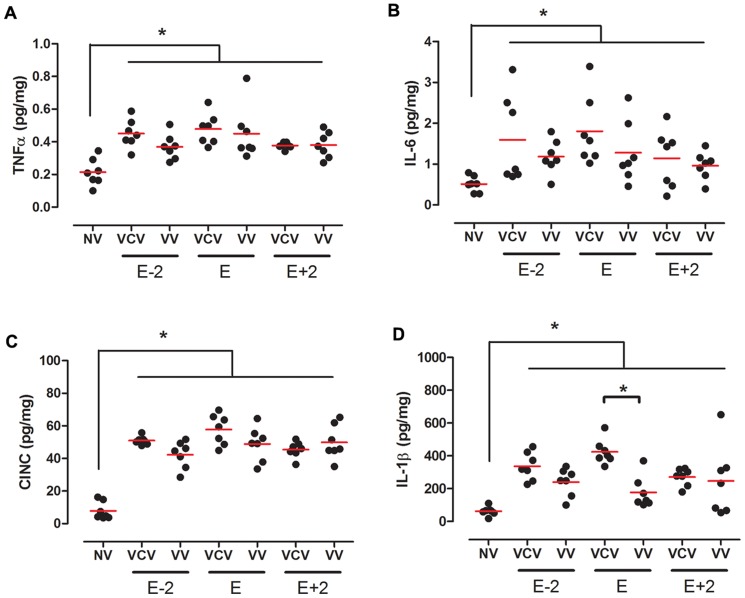
Diffuse alveolar damage score. Score of 0–9 to alveolar edema (A), interstitial edema (B), hemorrhage (C), inflammation (D), atelectasis (E) and the cumulative total score in the groups: VCV_E (volume-controlled mode and PEEP_minErs_; n = 6), VCV_E+2 (volume-controlled mode and PEEP_minErs_ plus 2 cmH_2_O; n = 6), VCV_E-2 (volume-controlled mode and PEEP_minErs_ minus 2 cmH_2_O; n = 6), VV_E (variable ventilation and PEEP_minErs_; n = 6), VV_E+2 (variable ventilation and PEEP_minErs_ plus 2 cmH_2_O; n = 5), VV_E-2 (variable ventilation and PEEP_minErs_ minus 2 cmH_2_O; n = 6). Red lines represent mean values. Ventilatory mode and PEEP effects were tested with two-way ANOVA followed by Tukey post hoc. T-test was used to detect differences between ventilated and non-ventilated groups and then correction by multiple comparison. Statistical significance was accepted at p<0.05. *p<0.05.

Oxygenation and gas exchange data are shown in Table 2. PaO_2_/FiO_2_, PaCO_2_ and pH did not differ either among groups or with PEEP, ventilatory mode and time. All mechanically ventilated animals presented a significant reduction in the air fraction in dorsal and basal regions, which means that collapse, edema or consolidation are concentrated in these areas, independently of ventilatory mode or PEEP level (Fig. 4A). Additionally, no significant differences were observed in the cumulated diffuse alveolar damage score among groups (Fig. 5).

**Table 2:** Gas exchange.

Group	Time	pH	PaO_2_/FiO_2_	PaCO_2_ (mmHg)
**VCV_E-2**	**5 min**	7.27±0.05	345±117	58±12
	**120 min**	7.25±0.06	395±144	56±7
**VV_E-2**	**5 min**	7.34±0.03	297±64	47±6
	**120 min**	7.30±0.05	367±53	54±10
**VCV_E**	**5 min**	7.32±0.03	270±122	49±10
	**120 min**	7.24±0.04	276±126	55±8
**VV_E**	**5 min**	7.30±0.02	319±52	56±5
	**120 min**	7.23±0.06	370±68	58±10
**VCV_E+2**	**5 min**	7.26±0.04	348±127	60±6
	**120 min**	7.20±0.06	420±119	58±8
**VV_E+2**	**5 min**	7.30±0.04	283±63	54±8
	**120** **min**	7.23±0.07	294±127	52±20

Values are expressed by mean ± SD. Definition of abbreviation: VCV_E: volume-controlled mode and PEEP_minEr_; VCV_E+2: volume-controlled mode and PEEP_minErs_ plus 2 cmH_2_O; VCV_E-2: volume-controlled mode and PEEP_minErs_ minus 2 cmH_2_O; VV_E: variable ventilation and PEEP_minErs_; VV_E+2: variable ventilation and PEEP_minErs_ plus 2 cmH_2_O; VV_E-2: variable ventilation and PEEP_minErs_ minus 2 cmH_2_O. Differences between groups were tested with Two-way ANOVA. Statistical significance was accepted at p<0.05.

**Figure 6:**
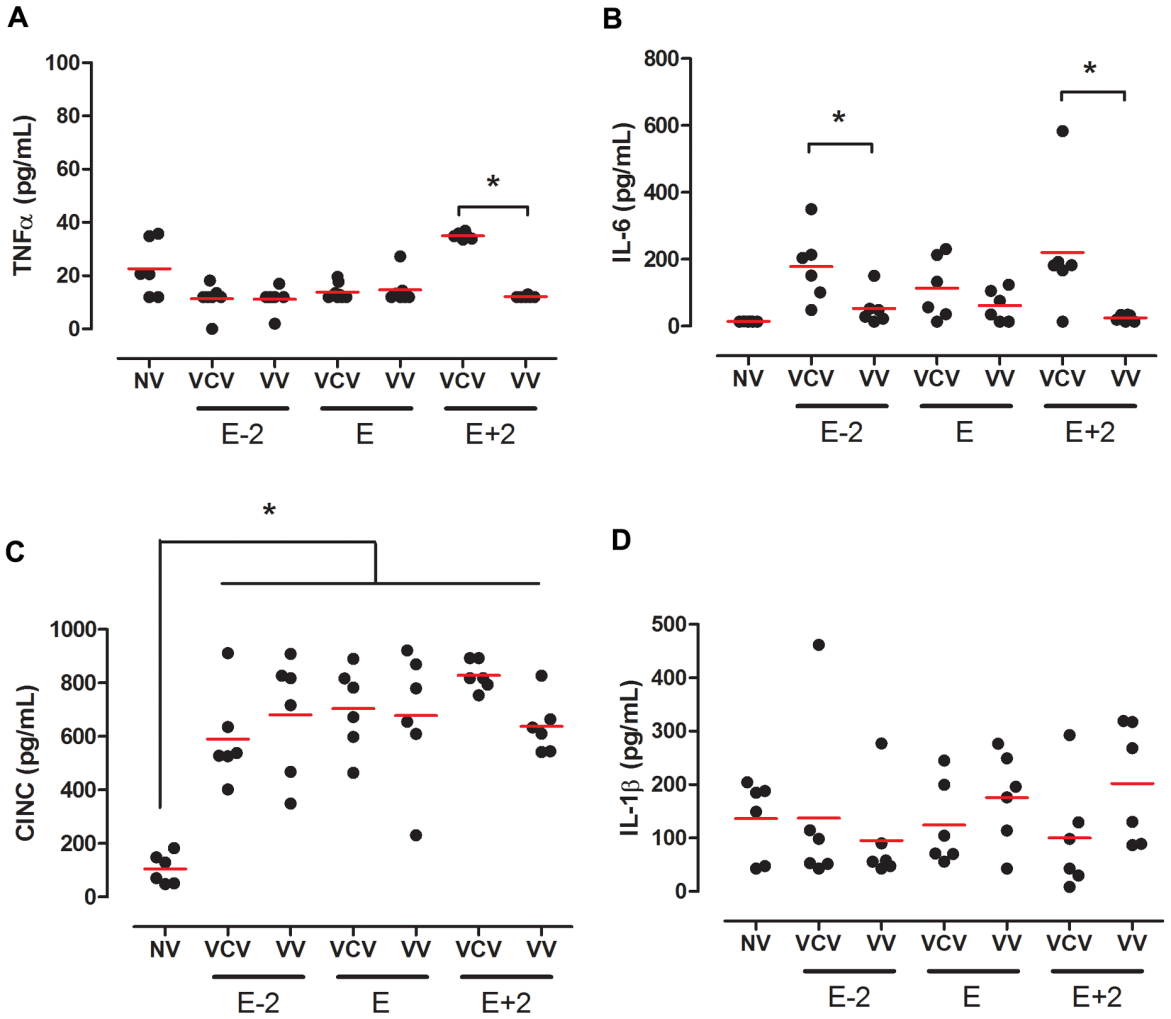
Lung cytokines. Lung tissue concentration of TNF-α (A), IL-6 (B), CINC-1(C) and IL-1β(D) in the groups: VCV_E (volume-controlled mode and PEEP_minErs_; n = 6), VCV_E+2 (volume-controlled mode and PEEP_minErs_ plus 2 cmH_2_O; n = 6), VCV_E-2 (volume-controlled mode and PEEP_minErs_ minus 2 cmH_2_O; n = 6), VV_E (variable ventilation and PEEP_minErs_; n = 6), VV_E+2 (variable ventilation and PEEP_minErs_ plus 2 cmH_2_O; n = 5), VV_E-2 (variable ventilation and PEEP_minErs_ minus 2 cmH_2_O; n = 6). Red lines represent mean values. Ventilatory mode and PEEP effects were tested with two-way ANOVA followed by Tukey post hoc. T-test was used to detect differences between ventilated and non-ventilated groups and then correction by multiple comparison. Statistical significance was accepted at p<0.05. *p<0.05.

The concentrations of lung tissue pro-inflammatory interleukins were higher in ventilated groups than in non-ventilated controls (Fig. 6). At the level of PEEP_minErs_, VV had significant less IL-1β than VCV (177±96 vs 424±76 pg/mg, respectively; p<0.001).

**Figure 7:**
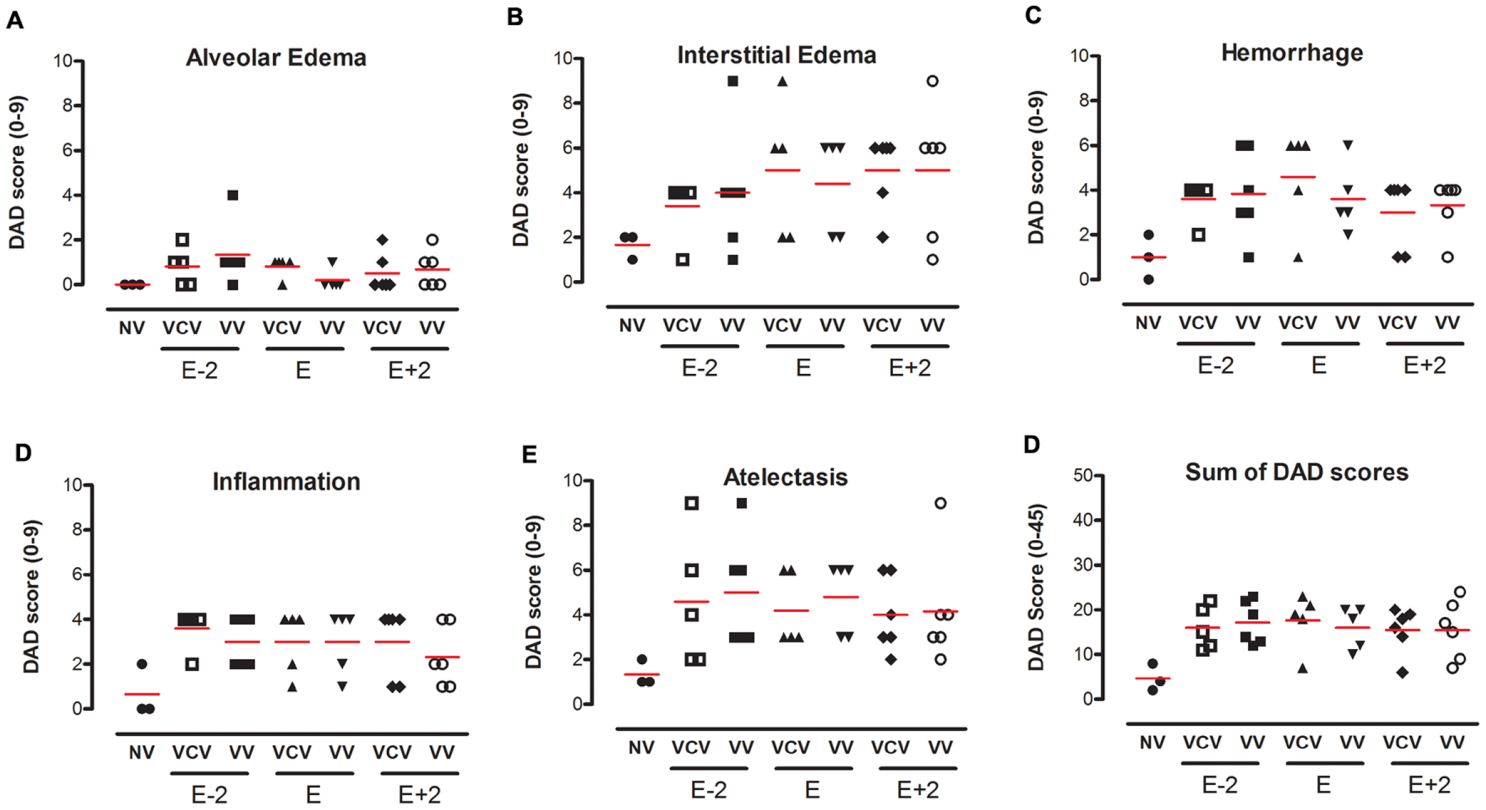
Plasmatic cytokines. Plasmatic concentration of TNF-α (A), IL-6 (B), CINC-1(C) and IL-1β (D) ) in the groups: VCV_E (volume-controlled mode and PEEP_minErs_; n = 6), VCV_E+2 (volume-controlled mode and PEEP_minErs_ plus 2 cmH_2_O; n = 6), VCV_E-2 (volume-controlled mode and PEEP_minErs_ minus 2 cmH_2_O; n = 6), VV_E (variable ventilation and PEEP_minErs_; n = 6), VV_E+2 (variable ventilation and PEEP_minErs_ plus 2 cmH_2_O; n = 5), VV_E-2 (variable ventilation and PEEP_minErs_ minus 2 cmH_2_O; n = 6). Red lines represent mean values. Ventilatory mode and PEEP effects were tested with two-way ANOVA followed by Tukey post hoc. T-test was used to detect differences between ventilated and non-ventilated groups and then correction by multiple comparison. Statistical significance was accepted at p<0.05. *p<0.05.

Plasmatic cytokines levels are presented in Figure 7. TNF-α and IL-6 concentration were similar among groups (Figs. 7A to D). We observed that CINC-1 levels were uniformly higher in ventilated groups than in non-ventilated controls (Fig. 7C) and IL-6 was lower in VV than in VCV at E-2 and E+2 PEEPs.

## Discussion

Patients under general anesthesia develop a progressive deterioration of lung function [34], with impaired oxygenation and lung compliance [3], [7]. Anesthesia-induced atelectasis might create a hazardous mechanical stress concentration in the neighborhood of atelectatic regions [35]. Such stress can predispose epithelial disruption and loss of function of the alveolar-capillary barrier leading to increased epithelial permeability. As a consequence, alveolar edema that dilutes and/or inactivates surfactant may further aggravate atelectasis [36].

Mechanical ventilation at the operating room has aimed at optimizing gas exchange in spite of lung protection [37]. Protective ventilatory strategies have just recently been applied to general anesthesia in lung-healthy patients [21], [38]. Most often, high tidal volumes and low or even no PEEP [39] associated with high inspiratory oxygen fraction are used [7], [40]. Even if airway pressure is limited, such ventilatory strategy might generate tidal hyperinflation of previously normally aerated areas (volutrauma), cyclic alveoli and small airways derecruitment (atelectrauma), and increase the levels of local proinflammatory mediators without ultrastructural damage (biotrauma) [41].

Minimization of injurious mechanical forces is the main target of protective ventilatory strategies that help to avoid lung collapse and overdistension by the use of low V_T,_ limitation of the plateau pressure, PEEP, and recruitment maneuvers. However, even during protective low V_T_ strategies, injurious mechanical stress can derive from concentration of stress in the interface between atelectatic and aerated regions [35], propagation of air/fluid interfaces in the airway producing injury on the bronchiolar epithelium [42], and energy release by the rupture of liquid bridges during airway reopening [43].

In the present study, we hypothesized that the combination of a moderate PEEP [19] and VV can be useful at the scenario of general anesthesia as an alternative to ventilatory strategies associated with high PEEP levels.

Our main findings were: 1) a progressive increase in Ers independent of ventilatory mode or PEEP was observed in lung-healthy rats during general anesthesia and muscle paralysis; 2) the rates of rise in Ers as well as lung tissue IL-1β concentration were significantly lower when VV was combined with the PEEP adjusted at the level of minimum Ers; 3) the same rate of increase in Ers observed during VV and PEEP of minimum Ers could only be achieved with higher levels of PEEP in the VCV_E+2 group; 4) even with a protective low-V_T_ strategy all ventilated animals presented higher levels of pro-inflammatory cytokines in lung tissue samples, independently of ventilatory mode and PEEP level, than non-ventilated animals.

A substantial increase in Ers was observed in all animals, suggesting a progressive alveolar derecruitment induced by anesthesia (Figure 3-A, -B, and -C), as previously suggested [44], [45]. Fairly higher levels of PEEP, ranging from 5 to 7 cmH_2_O, were required to minimize the rate of increase of Ers (Table 1). If we consider that the mean transpulmonary pressure in a rat at functional residual capacity ranges from 2 to 3 cmH_2_O [46], a PEEP of 7 cmH_2_O represents approximately a 2.3-fold increase. If the mean transpulmonary pressure in humans at functional residual capacity is about 5 cmH_2_O, our PEEP of 5–7 cmH_2_O in rats might correspond to a PEEP ranging from 11 to 16 cmH_2_O in humans. Such PEEP levels seem much higher than the standard of care at the operating room for lung-healthy mechanically ventilated patients [21].

Interestingly, the rate of increase in Ers fell when VV was combined with a PEEP at the level of the minimum Ers (ranging from 3 to 4 cmH_2_O in the present study, Table 1). If the same aforementioned conversion factor were applied, this PEEP might correspond to 7–9 cmH_2_O in lung-healthy patients. Such PEEP levels have been applied as a protective ventilatory strategy in patients undergoing abdominal surgery [21].

Several studies report a better performance of VV than VCV in ARDS experimental models in terms of mechanical function, pro-inflammatory biomarkers [44] and gas exchange [47]. From the mechanistic point of view, the dynamics of recruitment and de-recruitment of some alveolar units are likely to be important determinants of the efficiency of VV [45].

In the anesthesia scenario, VV was applied in a trial with 41 patients undergoing prolonged anesthesia for elective abdominal surgery. A sustained improvement in gas exchange was observed [30]. The benefits of protective low V_T_ ventilation in patients with ARDS, as well as the use of high levels of PEEP is well studied, but it is still under discussion whether these strategies should also be applied to lung-healthy patients in the anesthesia scenario. Additionally, considering that Ers changes overtime, the PEEP adjusted just after the recruitment maneuver, may not be able to keep lung opened throughout the protocol. In this scenario, VV ventilation can be an aid in lung stabilization.

In our study even though all experimental groups were ventilated with a protective strategy with respect to V_T_, they presented concentrations of TNF-α, IL-6, CINC-1 and IL-1β in lung homogenate higher than non-ventilated rats, but specifically IL-1β content showed a reduction on VV strategy (Figure 3).

Mechanical ventilation is likely to induce the production and/or secretion of IL-1β in animal models of VILI [48], [49]. Since IL-1β is a proinflammatory cytokine and a mediator of sterile inflammation that acts through IL-1R, it may have a role in the mechanism of lung inflammation and injury induced by mechanical stretch [49]. Additionally, activation of the inflammatory response, including increased IL-1β signaling, is a major mechanism of alveolar barrier dysfunction in VILI [48].

Plasmatic concentrations of IL-1β in all groups were compatible with non-ventilated controls. The amount of this protein in the lungs is about four hundred times that in the blood, showing that with 2 hours of ventilation the inflammatory process is mostly confined to the lungs. Our data showed that plasmatic levels of IL-6 were significantly higher in VCV strategies (VCV_E-2 and VCV_ E+2) compared to non-ventilated controls.

Pecchiari et al. recently showed that 4-h mechanical ventilation with different ventilatory modes did not alter lung histology, but exerted distinct effects on pro-inflammatory cytokine concentrations in lung-healthy rats [50]. These results are in agreement with our findings describing differences in cytokine concentration but not in lung histology. A similar behavior was also described by Krebs et al. ventilating lung-healthy rats during 1, 6 or 12 h following the open lung approach [51]. These authors found alterations in pulmonary histology just after 12 h of MV, whereas mRNA expressions of pro-inflammatory and pro-fibrotic cytokines were already elevated at 1 or 6 h of mechanical ventilation. Ventilation with the least injurious volume settings induced biochemical and histological changes consistent with lung impairment in a murine VILI model [52]. It triggered significant gene expression (including those involved in immunity and inflammation such as IL-1β and IL-6) at only 90 min of protective ventilation in the absence of a primary pulmonary insult in rodents [53].

## Limitations

Our findings around the amount of air/non-air expressed by air fraction obtained by histological analysis revealed no differences between VV and VCV, nor in PEEPs. This can be partially attributed to the fact that all lungs were removed under no PEEP. In fact, in absence of PEEP, an immediately collapse takes place and this probably contributed to an increase in the non-air fraction in all groups. However, the decision to excise the lungs with no PEEP was based on the technical difficulty to keep alveolar pressure constant during lung removal.

Another important issue is that in VV we applied a coefficient of variation (CV) of 10% to VT, while most studies use a CV of 30%. This can explain the similar oxygenation or CO_2_ clearance found in VCV and VV. However, the CV of 30% would generate volumes in the range 2–12 ml/kg, which is not recommended as a protective strategy.

Finally, mechanical ventilation was provided for just 2 h in all ventilated animals. This seems to be a short time period for the observation of any conclusive inflammatory or anti-inflammatory pathway. However, this is the most common period of mechanical ventilation during anesthesia, and only in few cases anesthesia and mechanical ventilations takes longer than this time window [38]. Furthermore, despite no significant differences in terms of oxygenation among ventilatory strategies, the lower concentration of IL-1β in lung tissue in the group of VV and PEEP adjusted at the minimal Ers may suggest a lower biological cost that might impact in a long term ventilation.

## Conclusion

VV with moderate levels of PEEP was associated with improved pulmonary function and alveolar stability in lung-healthy rats under general anesthesia.
